# Wishful Thinking

**Published:** 1987-11

**Authors:** J. L. Burton

**Affiliations:** Consultant Dermatologist, Bristol Royal Infirmary


### Wishful thinking

**J. L. Burton**
Consultant Dermatologist, Bristol Royal Infirmary

*Scene:* The Out-patient Clinic, St. Elsewhere's, 1988.
*Consultant:* . . . . . just keep taking the tablets, Mrs Jones, and we will write to you when there is a bed available . . . . . yes I know it's painful, and six months *is* a long time to wait, but I am afraid we have already lost two wards this year because of the cuts. Mrs Thatcher keeps taking money away from us to spend on Trident Missiles . . . . . Yes I wish you *would* write to your Member of Parliament about it. Hello Sister, is it tea time yet?
*Sister:* Not yet I'm afraid. There's another 14 to see before tea, but the Unit Manager wants a word with you first. He's come to explain the new administrative system to you.
*Consultant:* Well thank God somebody understands it. Send him in will you?

*20 minutes later:*
*Unit Manager:* . . . . . so what it comes down to, is that each Consultant is to get three wishes fulfilled throughout his career, but I must stress that the new rules are quite specific on this point, its three wishes and three wishes only during the whole time of your employment by the N.H.S. It seems a very fair and sensible system to me. All Consultants will be treated equally, they will all get exactly what they ask for, and it will save a considerable amount of time in Committees. The wishes may of course be expressed at any time before retirement and will be fulfilled forthwith. You can summon me immediately by bleep, or if that fails just rub this dusty old ophthalmoscope.
*Consultant:* What a superb idea, I'll make my first wish right away please.
*The Unit Manager smiles graciously.*
*Consultant:* (breathlessly) Well first of all I wish for a real tea-break in the middle of the clinic, with salmon and cucumber sandwiches served on a china plate, and chocolate cake, Oh, and a pot of real tea with a jug of fresh milk please, and no more of that powdered horse-pee that came out of that automatic dispenser I had to queue to use yesterday. Oh, and I would like it served by a proper old-fashioned nurse please, you know, a pretty one with a small waist and a silver buckle and a frilly cap and black stockings with, um . ., er . . a seam up the back.
*Unit Manager:* Immediately sir. I don't think this will need to go to Committee. We shall start with the afternoon clinic.
*The afternoon tea-break:* The Consultant is munching a salmon sandwich, and is chatting to a beautiful nurse, who gazes at him in starry-eyed admiration. There is a discreet knock at the door and the Unit Manager appears.
*Unit Manager:* Is everything to your satisfaction sir?
*Consultant:* Absolutely excellent thanks. These sandwiches are first rate. What a splendid system this is. Much better than all that consensus nonsense. I must say I think Maggie deserves to get in again.
*Unit Manager:* I'm so pleased sir. Just let me know when you are ready for your second wish.

*The same clinic, two weeks later:* Consultant opens pay-cheque, mutters and rubs ophthalmoscope vigorously. The Unit Manager manifests himself immediately.
*Unit Manager:* (smiling knowingly) I obey your summons sir. Are you ready for your second wish now?
*Consultant:* I should say I bloody well am. Look at this damned pay-cheque! £150 per week deducted at source for Refreshment and Entertainment. I wish to have my salary restored to normal immediately, and that goes for all subsequent pay-cheques.
*Unit Manager:* It shall be done immediately, your Omnipotence. Please let me know when you are ready for your third wish, but I feel I should perhaps remind you that this will be your final wish before retirement. I expect you will want to give careful thought to the needs of your Department, and the service it provides, perhaps after discussion with some of your colleagues.
*Consultant:* (frostily). I hardly think that will be necessary, thank you. I will make my third wish right away if you don't mind.
*Unit Manager:* If you insist, but don't say I didn't warn you.
*Consultant:* I wish that the regulations be changed so that in future all my wishes will be fulfilled immediately.

*There is a prolonged silence during which the Unit Manager begins to sob quietly.*
*Consultant:* There, there, don't take it too badly. I might get less demanding as I get older. Some Consultants do, you know.
*Unit Manager:* (Dabbing his eyes and trying to smile bravely). I fear that a Consultant who is already such an accomplished manipulator is hardly likely to improve with age, if I may say so.
*Consultant:* Me, manipulative? You were the one who tried to buy my silence with all this three wishes and three wishes only nonsense. Anyway I'm afraid I must go home now and mow my lawn. Before you vanish though, I wish you would employ some more nurses and some proper cleaners and reopen all the wards immediately. I also wish you would leave an experienced Senior Registrar to finish this clinic off for me, and I would like a new car to be provided by the N.H.S.—a Porsche please.
*The Unit Manager nods dejectedly, claps his hands and the Senior Registrar materializes.*
*Unit Manager:* The car's outside.
*He hands the Consultant his gold-headed cane.*
*Unit Manager:* Before you go, will you tell me one thing? Why did you lead me on with those first two wishes?
*Consultant:* It's quite simple really. If, for my first wish, I'd expressed the wish for all my subsequent wishes to be fulfilled, I'd have wasted two perfectly good wishes wouldn't I? I'd have thought that was fairly obvious, even to an Administrator.
*Unit Manager:* Bastard.
*Consultant:* Vanish!

